# The Energy Mix Concentration Index (EMCI): Methodological considerations for implementation

**DOI:** 10.1016/j.mex.2019.05.023

**Published:** 2019-05-23

**Authors:** Mar Rubio-Varas, Beatriz Muñoz-Delgado

**Affiliations:** a Institute for Advanced Research in Business and Economics (INARBE), Universidad Pública de Navarra (UPNA), Spain; b Department of Economic Analysis: Economic Theory and Economic History, Universidad Autónoma de Madrid (UAM), Spain

**Keywords:** Herfindahl-Hirschman Index, Energy mix, Energy consumption, Energy concentration, Energy diversification, Energy security, Energy transition, Decarbonization, Herfindahl-Hirschman Index, Energy history

## Abstract

The Energy Mix Concentration Index (EMCI) is a quantitative indicator of concentration of the energy mix based upon the Herfindahl-Hirschman Index. We use the EMCI to compare the evolution of the diversification (versus concentration) of energy mixes in the long-term in order to reveal the transformations of the energy structures which determine energy transitions. In this methodological paper we make explicit how to aggregate the energy sources in order to calculate the EMCI, including questions of detail such us the level of aggregation and the transformation of primary electricity to add it up to total consumption. We present alternative figures that illustrate some additional aspects of the relation of the EMCI to total consumption, consumption per capita and energy annual growth. We also show the sensitivity of the indicator to alternative specifications (with and without pre-modern energy sources) and alternative data sets, proving its robustness.

• Indicate how to aggregate energy carriers in the calculation of a quantitative index of concentration of the energy mix.
• Compare alternative specifications (with or without pre-modern energy carriers).
• EMCI focus on the major energy sources in the energy systems.

## Graphical abstract

**Figure:**
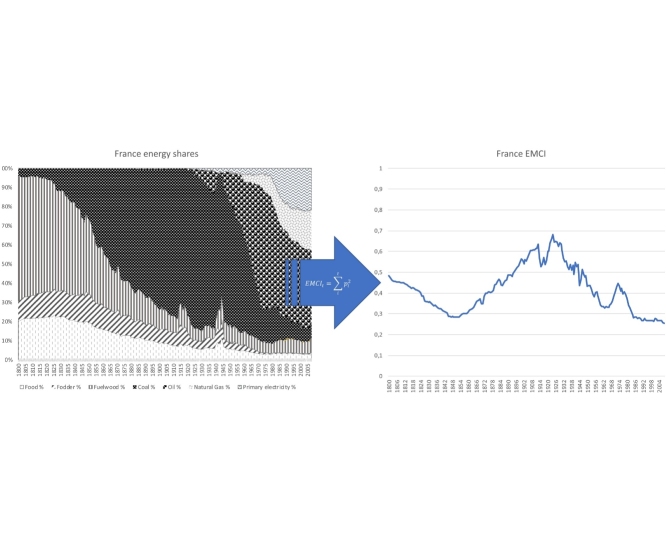

**Specifications Table**

**Table:** 

**Subject Area:**	*Energy*
**More specific subject area:**	*Energy economics, energy transition, energy mix*
**Method name:**	*Herfindahl-Hirschman Index*
**Name and reference of original method:**	*The paternity of this index is shared by economists Orris C. Herfindahl and Albert O. Hirschman. In 1945, Hirschman (in National Power and the Structure of Foreign Trade. University of California Press,) proposed an index of trade concentration consisting of the square root of the sum of the squares of the market share of each country in the market. For his part, in 1950, Herfindahl (in his doctoral dissertation, Concentration in the steel industry, Columbia University) proposed an index for measuring the firms’ concentration in the steel industry, which was computed in the same way as the Hirschman index, but without the square root that is, the sum of squares of firm sizes, all measured as percentages of total industry size. In Hirschman* [7] *he claimed the authorship of the index.*
**Resource availability:**	*The raw data on energy use is available at:*http://www.fas.harvard.edu/∼histecon/energyhistory/energydata.html*Alternative data for the United Kingdom can be retrieved from: Fouquet, R. (2014) ‘Long run demand for energy services: income and price elasticities over 200 years.’ Review of Environmental Economics and Policy 8(2) 186-207.*

## Method details

**Fig. 1:**
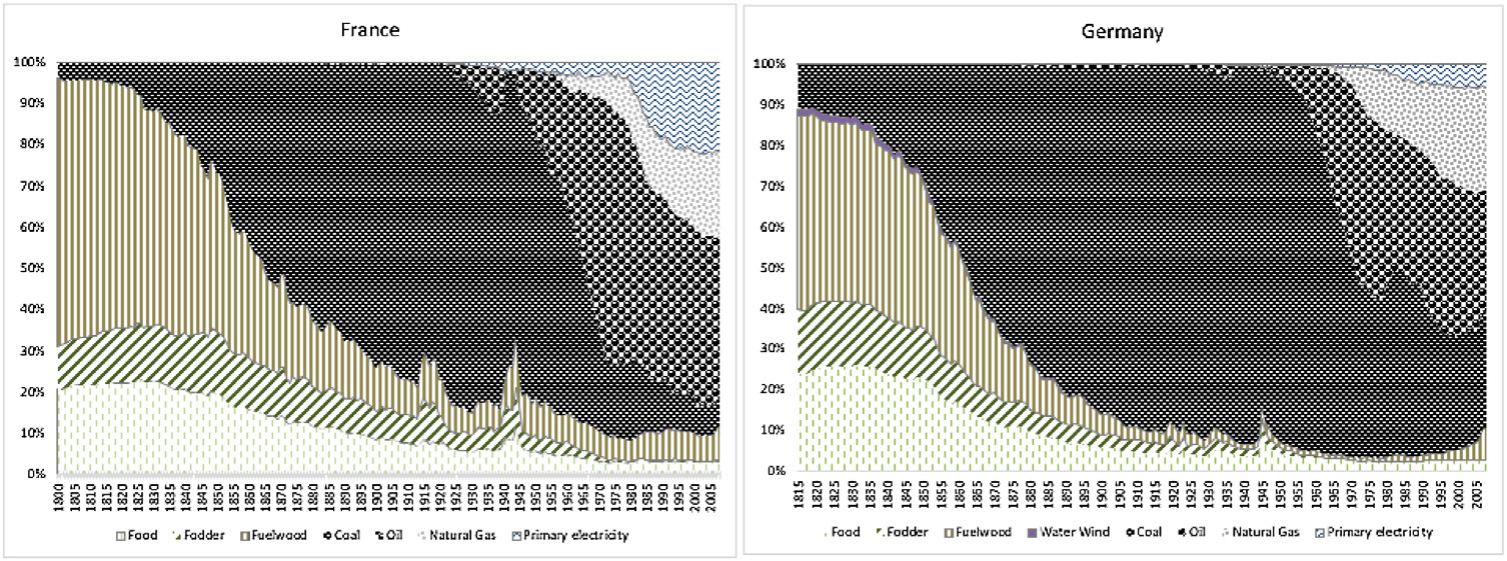
Shares of energy consumption in France and Germany 1800–2008 (%).

Even though transformations of the energy structure determine energy transitions, the diversification of energy mixes per se has not been studied from a long-term comparative perspective, making use of concentration indicators. The issue at stake is to offer an indicator that allows a synthetic comparison of the concentration of the distinct energy mixes of two or more countries, as for example the two shown in Fig. 1. For the naked eye it is impossible to stablish a ranking of which of these countries endure a more concentrated energy mix or its evolution over time.

Sources and notes: Elaborated by the authors with the data in Kander et al. [2], available at https://sites.fas.harvard.edu/˜histecon/energyhistory/energydata.html.

In Rubio-Varas and Muñoz-Delgado [1] we propose the use of the Energy Mix Concentration Index (EMCI) as a quantitative index of concentration of the energy mix based upon the Herfindahl-Hirschman Index (HHI).^1^ Here we offer some methodological considerations for its implementation in the future to replicate our results and in order to use the EMCI to other country cases.

The HHI commonly applies to market concentration analysis. It is measured by the sum of the squares of the market shares of each energy source in any given period, which corresponds to the formula:

(1) $${HHI}_{t}=\sum_{i}^{t}p_{i}^{2}$$

where *p_i_* is the share of the energy source *i* in the energy mix. Smaller values of the HHI indicate greater diversification, with 0 being the minimum concentration and 1 being the maximum concentration (in case the shares are expressed as fractions, where the aggregation of all the portions sum one i.e., 10% would be considered as 0.1).^2^ Accordingly, the concentration of the energy mix in a given year has been calculated using the HHI, and we named it Energy Mix Concentration Index (EMCI). For each country, we built a matrix containing the share of each energy source in the total energy consumption of every year from 1800 to 2010, for selected countries (i.e. United Kingdom, France, Germany, and the Netherlands, Italy, Portugal, Spain, and Sweden).^3^

For our calculations the energy sources are aggregated at the upper level possible following the classification originally made in Kander et al. [2]: food, fodder, firewood (biomass), direct water/wind/sun (prime movers or heaters), coal, oil, natural gas and primary electricity (from hydro, geothermal generators, wind power, photovoltaic and nuclear power). Thus rewriting Eq. (1) becomes:

(2) $${EMCI}_{t}=\sum_{i}^{t}p_{i}^{2}$$

Where *i* are:

• Food
• Fodder
• Wind & water & sun (direct use for prime movers and heaters only)
• Firewood & biomass
• Coal
• Oil
• Natural Gas
• Primary electricity (at heat value equivalent from all renewable sources plus nuclear)

Next we show one empirical example with the data of France, step by step:

1) Download the data, which in this case is already aggregated at the upper possible level of energy carrier as shown in Table 1.

**Table 1:** Sample of raw data (French energy shares).

	Food	Fodder	Firewood	Water	Coal	Oil	Natural Gas	Primary electricity
				Wind				
	%	%	%	%	%	%	%	%
**1800**	20,5	10,4	65,4	0	3,8	0	0	0
**1801**	20,6	10,6	64,7	0	4,2	0	0	0
**1802**	20,9	10,7	63,8	0	4,6	0	0	0
**1803**	21,3	10,9	63,3	0	4,5	0	0	0
**1804**	21,7	11	62,9	0	4,4	0	0	0
**1805**	21,6	11,1	62,8	0	4,4	0	0	0
**1806**	21,8	11,2	62,6	0	4,4	0	0	0
**1807**	21,7	11,4	62,6	0	4,4	0	0	0
…	…	…	…	…	…	….	…	…
2) Calculate the square of each of the shares
3) Sum and normalise to 1 (this last step is optional), the results for the sample raw data are shown in Table 2.

**Table 2:** EMCI sample calculation for French data.

	Food	Fodder	Firewood	Water	Coal	Oil	Natural Gas	Primary electricity	EMCI
				Wind					Sum sq
	sq share	sq share	sq share	sq share	sq share	sq share	sq share	sq share	normalised to 1
**1800**	420,25	108,16	4277,16	0	14,44	0	0	0	0,482001
**1801**	424,36	112,36	4186,09	0	17,64	0	0	0	0,474045
**1802**	436,81	114,49	4070,44	0	21,16	0	0	0	0,46429
**1803**	453,69	118,81	4006,89	0	20,25	0	0	0	0,459964
**1804**	470,89	121	3956,41	0	19,36	0	0	0	0,456766
**1805**	466,56	123,21	3943,84	0	19,36	0	0	0	0,455297
**…**	…	…	…	…	…	…	…	…	…

In the Appendix A we provide the graphical results of the EMCI for the 8 European countries analysed in Rubio-Varas and Muñoz-Delgado [1], against their level of total energy consumption, their energy use per square kilometres and their annual energy consumption growth. The remainder of this companion paper addresses a number of questions of detail and sensitivity analysis.

### Questions of detail: the level of aggregation and the transformation of primary electricity

In the example above the shares of each energy carrier were already calculated in the original source of the data. But more often, researchers will find the raw energy data and will need to aggregate it by themselves before calculating the share of each energy carrier. The criterion should always be to aggregate energy carriers at the upper possible level. Thus, for instance, the appearance of wood chips or other biomass carriers in recent years will not imply the addition of a different category with its own share. Instead, wood chips should engross the share of firewood since that is the upper level of aggregation of the major energy carriers.

Another important question at the time of aggregating energy is how to add up primary electricity. The classification of electricity as both primary and secondary energy commodity is used in the UN manual and the OECD/IEA/Eurostat manual. In the UN manual, electricity from nuclear, hydro, wind and geothermal sources is labelled as primary electricity. The OECD/IEA/Eurostat manual states that: “Electricity is produced as primary as well as secondary energy. Primary electricity is obtained from natural sources, such as hydro, wind, solar and tide and wave power. Secondary electricity is produced from heat of nuclear fission of nuclear fuels, from the geothermal heat and solar thermal heat and by burning primary combustible fuels such as coal, natural gas, oil and renewables and wastes”.

This distinction between thermal and non-thermal electricity generation is non-trivial for it has implications on how to add up the Kwh of electricity to the primary energy consumption, which in turn will have implications on the share of electricity on the overall primary energy consumption. For hydroelectric or wind (the principal non-thermal electricity generating technologies), one option could be to transform it in terms of the energy cost of producing the same amount of electricity thermally. That is, calculating how much coal / oil/natural gas/uranium would be necessary to produce a kilowatt-hour. Alternatively it can be considered that the hydro-electricity produced is, in itself, a primary energy form and simply calculate the caloric content of each kilowatt produced. The counterfactual option, that is, the hypothetical thermal cost has some notable disadvantages. In the first place, it is necessary to have the technical coefficients of transformation of each historical moment, that is, the efficiency with which the fossil mineral was converted into electricity. This would introduce a critical difference in the calculation with respect to the rest of energy resources in which the efficiency does not enter the calculation, only its calorific value.

Secondly, there is the paradox that using the counterfactual method, as we go back in time, the thermal plants were more inefficient. The more inefficient the thermal power plants the higher the primary energy consumption would appear, as more coal would have to have been burned to produce the same amount of electricity. In reality that coal never burned, so that energy never entered the productive system, the country could only make use of the amount of kilowatts generated in its waterfalls. In the same way, it would not make sense to use this transformation when estimating the country’s CO^2^ emissions or the polluting intensity of the energy used in the country, since that mineral never burned. These reasons have led to the decision of transforming the electricity generated in non-thermal plants as if it was primary energy, using the calorific value contained in the electricity generated.

For nuclear power, an obvious issue arises, since it is clearly a thermal process which produces electricity. International bodies such as the IEA transform nuclear electricity production using the ratio 1 Mwh = 0.2606 toe (tons of oil equivalent) while for the rest of the primary electricity production (hydroelectric, wind, wave and solar) the equivalence used is 1 Mwh = 0.086 toe. Put another way, each megawatt hour of nuclear power weights three times more the rest of megawatts in the estimation of a country's primary energy consumption. This impacts on other measures such as energy intensity (the primary energy ratio per unit of GDP), and the interpretation of the shares of the different energy sources. In the data provided by Kander et al. [2], nuclear is treated as a primary energy source and the nuclear generation transformed at its heat value, as the rest of the primary electricity generation.

### The EMCI without pre-modern energy sources & sensitivity analysis to different data sets

**Fig. 2:**
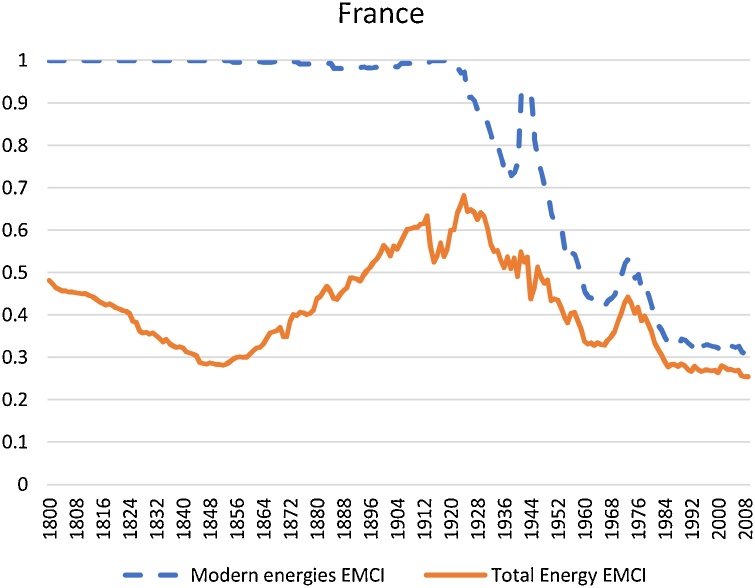
EMCI. The case of France, modern energies vs total energy.

Given the importance of traditional energy sources up to well into the 20th century, any attempt to measure the degree of concentration of the energy mix in the long run without including them will be flawed. Moreover, considering traditional energies is the only way to take into account the first energy transition, from organic-based energy sources to coal. Yet, we are aware that for implementing the EMCI in present day countries it would be costly to obtain estimations of pre-modern energy consumption (particularly for humans and draft animals). Besides they have a relative small importance nowadays. Thus, for illustration purposes, we also show the results of the EMCI applied only to modern energy carriers. In other words, one needs to recalculate the shares of each of the modern energy carriers on total modern energy consumption alone (that is the sum of coal, oil, natural gas and primary electricity but excluding pre-modern sources). Be aware that some biomass enters present day energy mixes but we are excluding firewood altogether in the estimates shown in Fig. 2.

Sources and notes: data from Kander et al. [2]. Modern energy refers to the sum of mineral coal, petroleum, natural gas, and the primary forms of generating electricity such hydroelectricity, nuclear energy, and renewable energies such as wind power, solar, geothermal, etc. Total energy adds to the mix the pre-modern sources (i.e. food for men and working animals, firewood, traditional wind and water used in wheels and mills, peat). EMCI measured by a HHI, as explained above. The smaller (larger) the EMCI, the more diversified (concentrated) the energy mix is.

Fig. 2 fully endorse our stand on the importance of traditional energy sources up to well into the 20th century in the degree of concentration of the energy mix in the long run. Yet for comparisons of the concentrations of the energy mixes after the 1970s in countries where the pre-modern energies play a residual role, it will be possible to implement the EMCI on modern carriers alone without losing much explanatory power.

**Fig. 3:**
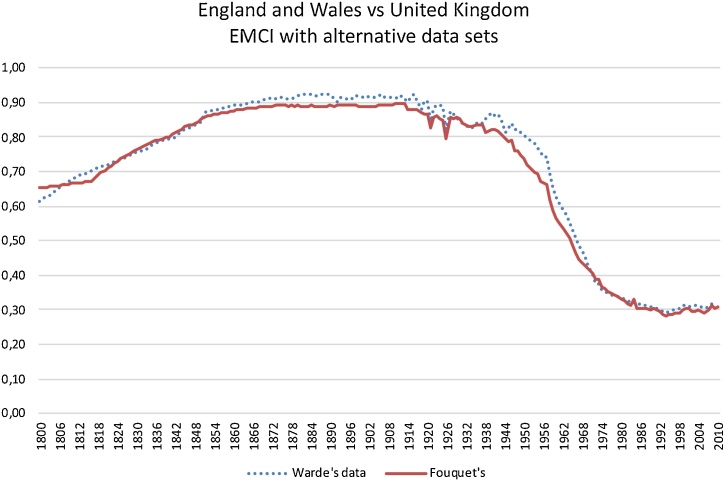
EMCI. The case of England and Wales vs United Kingdom.

A final consideration regarding the implementation of the EMCI in long-term series of data relates to its sensitivity to alternative data sets. There exist an alternative set of data for England and Wales by Warde [3] estimated independently from the one estimated for Fouquet [4] for the United Kingdom. The EMCI calculated over each of these two alternative data sets are shown in Fig. 3. They are very similar making evident the resilience of the ECMI to alternative datasets.

Sources and notes: England and Wales by Warde [3], Fouquet [4] for the United Kingdom. Both sets include pre-modern and modern energy sources (i.e., food for men and working animals, firewood, traditional wind and water used in wheels and mills, peat, mineral coal, petroleum, natural gas, and the primary forms of generating electricity such hydroelectricity, nuclear energy, and renewable energies such as wind power, solar, geothermal, etc.). EMCI measured by a HHI, as shown above. The smaller (larger) the EMCI, the more diversified (concentrated) the energy mix is.

## Figures and Tables

**Figure:**
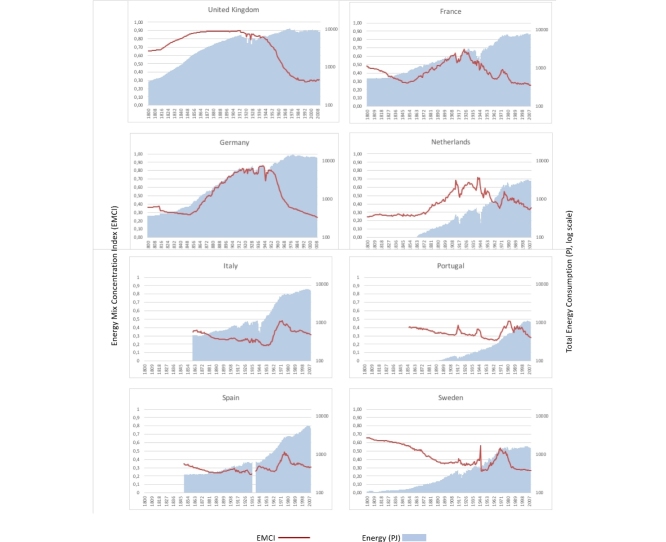


**Figure:**
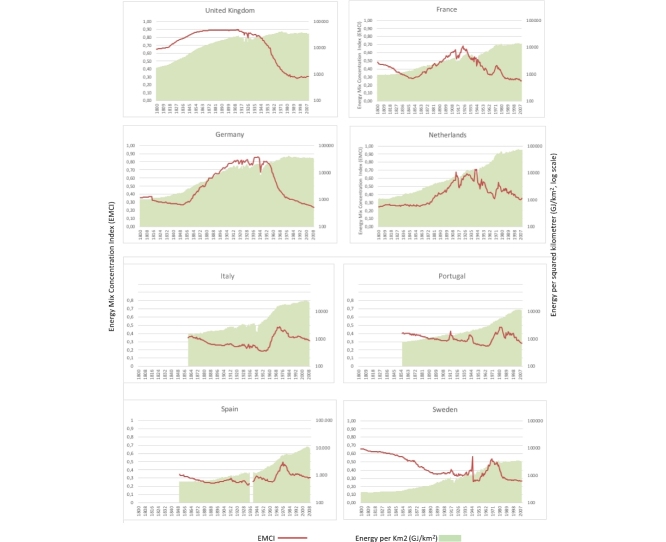


**Figure:**
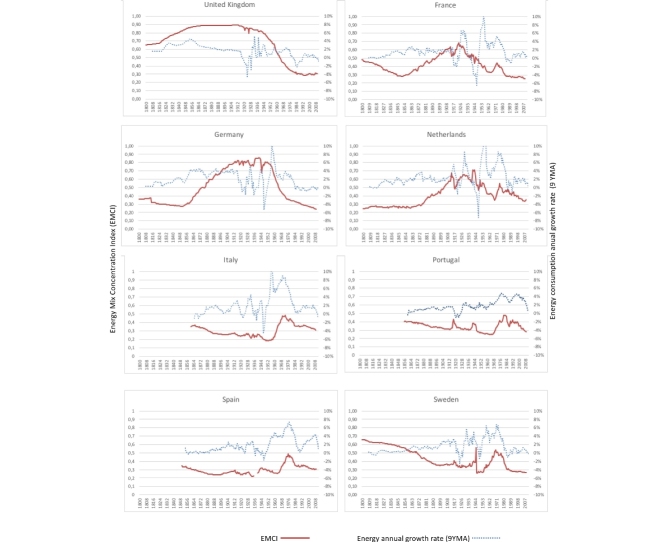


## Appendix A

Sources and notes: data from Kander et al. [2] except for the United Kingdom, which belongs to Fouquet [4], include pre-modern and modern energy sources (i.e., food for men and working animals, firewood, traditional wind and water used in wheels and mills, peat, mineral coal, petroleum, natural gas, and the primary forms of generating electricity such hydroelectricity, nuclear energy, and renewable energies such as wind power, solar, geothermal, etc.). EMCI measured by a HHI. The smaller (larger) the EMCI is, the more diversified (concentrated) the energy mix. Note the right axis logarithm scale.

Sources and notes: data from Kander et al. [2] except for the United Kingdom, which belongs to Fouquet [4], include pre-modern and modern energy sources (i.e., food for men and working animals, firewood, traditional wind and water used in wheels and mills, peat, mineral coal, petroleum, natural gas, and the primary forms of generating electricity such hydroelectricity, nuclear energy, and renewable energies such as wind power, solar, geothermal, etc.). EMCI measured by a HHI. The smaller (larger) the EMCI is, the more diversified (concentrated) the energy mix. Note the right axis logarithm scale.

Sources and notes: data from Kander et al. [2] except for the United Kingdom, which belongs to Fouquet [4], include pre-modern and modern energy sources (i.e., food for men and working animals, firewood, traditional wind and water used in wheels and mills, peat, mineral coal, petroleum, natural gas, and the primary forms of generating electricity such hydroelectricity, nuclear energy, and renewable energies such as wind power, solar, geothermal, etc.). EMCI measured by a HHI. The smaller (larger) the EMCI is, the more diversified (concentrated) the energy mix. Annual growth rates shown as nine years moving average (9YMA).

## Footnotes

1 The paternity of this index is shared by economists Orris C. Herfindahl and Albert O. Hirschman. In 1945, Hirschman [5] (in *National Power and the Structure of Foreign Trade*. University of California Press,) proposed an index of trade concentration consisting of the square root of the sum of the squares of the market share of each country in the market. For his part, in 1950, Herfindahl [6] (in his doctoral dissertation, *Concentration in the steel industry*, Columbia University) proposed an index for measuring the firms’ concentration in the steel industry, which was computed in the same way as the Hirschman index, but without the square root that is, the sum of squares of firm sizes, all measured as percentages of total industry size. In Hirschman [7] he claimed the authorship of the index.

2 Sometimes, the shares are expressed as percentages –i.e. 10% would be considered as 10–. In this case, the maximum concentration would be 10,000.

3 For Italy, Portugal and Spain, from mid-19th century.
